# PReS-FINAL-2248: Parovirus B19 associated unusual joint symptoms in twin girls

**DOI:** 10.1186/1546-0096-11-S2-P238

**Published:** 2013-12-05

**Authors:** H Sakalli, E Baskin, S Dener

**Affiliations:** 1 Pediatric Nephrology, Baskent University Faculty of Medicine, Konya, Turkey; 2 Pediatric Nephrology, Baskent University Faculty of Medicine, Ankara, Turkey; 3 Neurology, Baskent University Faculty of Medicine, Konya, Turkey

## Introduction

Human Parvovirus B19 (HPVB19) is the responsible agent of fifth disease. Other less common manifestations include transient aplastic crisis, nonimmune hydrops-fetalis, chronic arthritis, myocarditis, hepatitis, multisystemic vasculitis, renal disease, and idiopathic thrombocytopenic purpura. An increasing number of reports have described HPVB19 infection in association with a variety of neurologic manifestations. We described two cases of twin girls presented with the symptoms of carpal tunnel syndrome (CTS) associated with HPVB19 infection.

## Objectives: case reports

A previously healthy 6 year-old girl presented with sudden onset of swelling of the distal extremities associated with painful paresthesia of the first three digits of her both hands. 10 days earlier her parents had noticed an erythematous rash which spread from the face to the arms and abdomen. There were no histories of wrist trauma, recent immunisations or drug use. Physical examination revealed mild swelling of the metacarpophalangeal and proximal interphalangeal joints. She had mild hypoesthesia in the sensory dermatomes of both median nerves, a positive Tinel's sign on the right, and a mild decrease in thumb abduction bilaterally. The acute symptoms gradually subsided after 1 week of symptomatic treatment.

One week later her twin presented with sudden onset of swelling of the distal extremities associated with painful paresthesia of the last three digits of her both hands. Her medical history was similar. Physical examination showed bilateral hypoesthesia in the sensory regions of the both median and ulnar nerves associated with swelling of the hands. It was difficult and painful to close her hands and especially to open them. She had received the same treatments. The symptoms rapidly decreased within a few days, but numbness of the third and fourth digits persisted for 15 days.

## Methods: laboratory findings

In the first case, nerve conduction studies revealed moderate delay in distal sensory-motor latencies of both median nerves with delayed median nerve F-responses on both sides. In the second case, both median nerves sensory-motor conduction velocity at the wrist were slow and F-responses of both median and ulnar nerves were delayed bilaterally. In both cases, there were no denervation potentials and motor and sensory nerve conduction studies of peroneal, posterior tibial, and sural nerves and the posterior tibial F-responses were normal in both lower extremities. Screening blood tests including chemistry panel, complete blood count, erythrocyte sedimentation rate, C-reactive protein, rheumatoid factor, serum compleman 3 and 4, thyroid stimulating hormone, and antinuclear antibodies were normal in both cases. Rubella virus, Borrelia species, M. tuberculosis, and other Mycobacterium species were ruled out either clinically or laboratory tests.

## Results

Both patient's serological tests showed positive HPVB19 specific IgM and HPVB19-DNA was also detected by using PCR.

## Conclusion

Infectious agents, especially HPVB19 associated acute CTS should be considered among the patients presenting with acute joint findings in childhood.

## Disclosure of interest

None declared.

